# Disruption of Histone Modification and CARM1 Recruitment by Arsenic Represses Transcription at Glucocorticoid Receptor-Regulated Promoters

**DOI:** 10.1371/journal.pone.0006766

**Published:** 2009-08-26

**Authors:** Fiona D. Barr, Lori J. Krohmer, Joshua W. Hamilton, Lynn A. Sheldon

**Affiliations:** 1 Department of Physiology, Dartmouth Medical School, Lebanon, New Hampshire; 2 Department of Pharmacology & Toxicology, Dartmouth Medical School, Hanover, New Hampshire; 3 Center for Environmental Health Sciences, Dartmouth Medical School, Hanover, New Hampshire; Institute of Genetics and Molecular and Cellular Biology, France

## Abstract

Chronic exposure to inorganic arsenic (iAs) found in the environment is one of the most significant and widespread environmental health risks in the U.S. and throughout the world. It is associated with a broad range of health effects from cancer to diabetes as well as reproductive and developmental anomalies. This diversity of diseases can also result from disruption of metabolic and other cellular processes regulated by steroid hormone receptors via aberrant transcriptional regulation. Significantly, exposure to iAs inhibits steroid hormone-mediated gene activation. iAs exposure is associated with disease, but is also used therapeutically to treat specific cancers complicating an understanding of iAs action. Transcriptional activation by steroid hormone receptors is accompanied by changes in histone and non-histone protein post-translational modification (PTM) that result from the enzymatic activity of coactivator and corepressor proteins such as GRIP1 and CARM1. This study addresses how iAs represses steroid receptor-regulated gene transcription. PTMs on histones H3 and H4 at the glucocorticoid receptor (GR)-activated mouse mammary tumor virus (MMTV) promoter were identified by chromatin immunoprecipitation analysis following exposure to steroid hormone±iAs. Histone H3K18 and H3R17 amino acid residues had significantly different patterns of PTMs after treatment with iAs. Promoter interaction of the coactivator CARM1 was disrupted, but the interaction of GRIP1, a p160 coactivator through which CARM1 interacts with a promoter, was intact. Over-expression of CARM1 was able to fully restore and GRIP1 partially restored iAs-repressed transcription indicating that these coactivators are functionally associated with iAs-mediated transcriptional repression. Both are essential for robust transcription at steroid hormone regulated genes and both are associated with disease when inappropriately expressed. We postulate that iAs effects on CARM1 and GRIP1 may underlie some of its therapeutic effects and as well be associated with its toxic effects.

## Introduction

Chronic exposure to inorganic arsenic (iAs), in the most prevalent form of arsenite (As^3+^) from drinking water is one of the most significant and widespread environmental health risks in the U.S. and throughout the world [1]. Epidemiologic evidence links iAs exposure to an increased risk of lung, bladder, skin and other cancers, type 2 diabetes, vascular and cardiovascular disease, and reproductive and developmental anomalies [2], all of which can be linked to inappropriate steroid or nuclear receptor-mediated gene regulation which can have deleterious effects on every metabolic system and is associated with many forms of cancer [3]–[6].

Micromolar amounts of iAs inhibit transcription mediated by the glucocorticoid receptor (GR), the progesterone receptor (PR), the androgen receptor (AR), the estrogen receptor (ER) and the mineralocorticoid receptor (MR) [7]–[9], as well as the thyroid hormone (TR) and the retinoic acid (RAR) receptors [10]. This suggests an iAs target common to all or many nuclear receptor-regulated gene promoters but a mechanism has yet to be identified. Steroid hormone-regulated receptors belong to the superfamily of nuclear receptors that includes the GR, PR, ER, MR, and AR. All use similar transcriptional activation mechanisms to regulate physiological responses to a broad range of internal and external stimuli [11].

Following ligand binding, transcription by steroid receptors is initiated by receptor-DNA binding and changes in chromatin structure contributed to by changes in histone post-translational modifications (PTMs) [12]. Arsenic-associated changes in histone PTMs have been identified in transcriptional activation from some non-steroid regulated promoters [13] and global changes have been reported in response to iAs at histone H3 [14]. Histone PTMs, are regulated by coactivator or corepressor proteins [15] that interact with promoters via protein-protein interactions with the steroid receptor itself, or with other promoter-associated proteins. These co-regulatory proteins act as transducers between internal or external stimuli and a genetic response by providing targets for PTMs mediated by cell signaling pathways [16]. Coactivators such as CARM1 (coactivator-associated arginine methylatransferase) have enzymatic activities on histones and non-histone, promoter-associated proteins [17], [18]. CARM1 targets histone H3R17 and H3R26 for methylation upon activation of both ER and GR-regulated promoters [19], [20] and associates with these promoters by binding to one of the three p160 coactivators (SRC1, SRC2/GRIP1/TIF2, or SRC3/pCIP/AIB1/ACTR/RAC3) [20], [21] which in turn bind directly to the DNA-bound steroid receptor.

To understand how iAs represses steroid hormone-mediated gene transcription we sought to determine when in the transcription process an iAs effect could be detected and whether histone modification patterns changed in response to hormone alone compared to hormone plus iAs at the MMTV promoter. We found that iAs represses GR-mediated chromatin remodeling and transcription initiation and that methylation and acetylation at histone H3R17 and H3K18 respectively, decreased within minutes of iAs addition. Both of these histone PTMs are associated with transcriptional activation at steroid hormone-regulated promoters. Additionally, it was determined that CARM1 was absent from the promoter after treatment with iAs. Unexpectedly, while CARM1 may be a target for iAs, GRIP1 is also a probable target even though unlike CARM1 it was still associated with the promoter when cells were treated with iAs. Finally, the data suggest iAs-inhibited transcription is mediated through an indirect effect on one or both of these coactivator proteins that may be via deregulation of a cell signaling pathway.

## Results

### iAs Inhibits transcription from the GR-regulated MMTV Promoter

**Figure 1:**
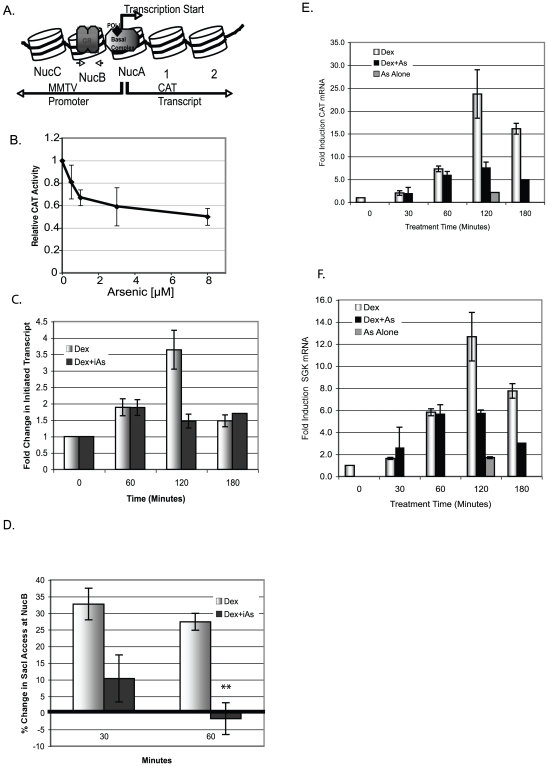
iAs inhibits transcription from the MMTV and SGK promoters. (A) MMTV promoter with Nucs A–C. Large arrow indicates transcription start site and small arrows, PCR primers in NucB. (B) CAT activity in 1470.2 cells treated (4 h) with 100 nM Dex±iAs. CAT activity expressed as a ratio relative to cells treated with 100 nM Dex alone (arbitrarily set at 1). *n = 3* replicates in a representative experiment. (C) Nuclear run-on analysis from the MMTV promoter in cells treated with 5 nM Dex±8 µM iAs for times indicated. Fold change = (CAT mRNA hybridized- background (pUC))/5s RNA) relative to basal transcription (no treatment) arbitrarily set to one. *n* = 3 replicates from two independent experiments. (D) REAA with restriction enzyme Sac1. SacI-digested DNA from cells treated for 30 or 60 min with 5 nM Dex±8 µM iAs indicates an “open” or “closed” chromatin template at MMTV NucB measured by qPCR with primers that span the SacI site in NucB. The zero value represents basal SacI digestion to which all treated values were compared. *n = 3* independent experiments. **P-value<0.01. (E & F) CAT or SGK mRNA measured by qRT-PCR. Cells treated with 5 nMDex±8 µM iAs for times indicated. Both (E&F) are data from the same experiment done in triplicate that is representative of 2 independent experiments.

To determine whether iAs inhibits GR-mediated transcription from a stably integrated MMTV-chloramphenicol acetyltransferase (MMTV-CAT) reporter gene, 1470.2 cells were treated with 100 nM Dex±iAs at a range of concentrations that can be found in drinking water. In contrast to a transiently expressed reporter gene, stably integrated MMTV-CAT is associated with histone proteins in a regularly spaced chromatin conformation [22] and resembles that of an endogenous gene (Fig. 1A). CAT activity was inhibited 20–50% with Dex plus 0.5 µM to 8 µM iAs compared to cells treated with Dex alone (Fig. 1B).

To determine whether iAs inhibited transcription at initiation or elongation cells were treated with 5 nM Dex±8 µM iAs and the amount of initiated CAT transcript from the MMTV promoter was determined by nuclear run-on analysis. Treatment with 5 nM Dex was used to slow the transcription process to enable evaluation of early events in activation. Dex alone increased transcript initiation with the peak at about 120 min and decreased initiation by 180 min, indicative of transcriptional repression (Fig. 1C). In contrast, treatment with Dex +8 µM iAs inhibited initiation of CAT mRNA. These data suggested that iAs inhibits transcription initiation. To test this further, restriction enzyme accessibility assays (REAAs) were done to determine whether iAs affects chromatin remodeling, an event associated with initiation. A Sac1 endonuclease cleavage site in the NucB region of the MMTV-CAT promoter is accessible after treatment with Dex but is less accessible before treatment and is an indicator of a GR-induced structural transition in the chromatin [23]. Treatment of cells with 5 nM Dex +8 µM iAs versus 5 nM Dex alone inhibits access to the Sac1 cleavage site by 30 minutes and by 60 minutes chromatin access decreases to less than or equal to basal levels (Fig. 1D). This suggests that the MMTV promoter shuts down progressively with time, in agreement with the nuclear run-on experiments where transcript is still associated with the promoter at 60 minutes but is not by 120 minutes when iAs is present. We do not view the seeming discrepancy in promoter accessibility and the presence of initiated transcripts at 60 minutes a problem because transcripts detected at 60 minutes would have initiated before the chromatin template was shut down and thus there should be a lag in when promoter access is inhibited and when transcript can be detected. Thus iAs inhibits transcription initiation and associated chromatin remodeling at the GR-regulated MMTV promoter.

Accumulated CAT mRNA was measured by qRT-PCR and by 2 hours there was significantly more CAT mRNA with Dex alone than with Dex plus 8 µM iAs (Fig. 1E), in agreement with the pattern of transcript initiation observed (Fig. 1C). Transcription at the endogenous GR-regulated serum glucocorticoid kinase (SGK) promoter was also inhibited by iAs (Fig. 1F) which indicates that the inhibitory effect of iAs on the stably integrated MMTV promoter recapitulates events on an endogenous promoter. Treatment with 8 µM iAs alone showed no change in the amount of CAT or SGK transcripts from background levels (Fig. 1E & F). Together, these data raised the possibilities that iAs may inhibit GR binding or stability at the glucocorticoid response element (GRE), or alternatively, the binding of another promoter-associated protein essential for initiation and activation.

### GR binds to promoter DNA in the presence of iAs

**Figure 2:**
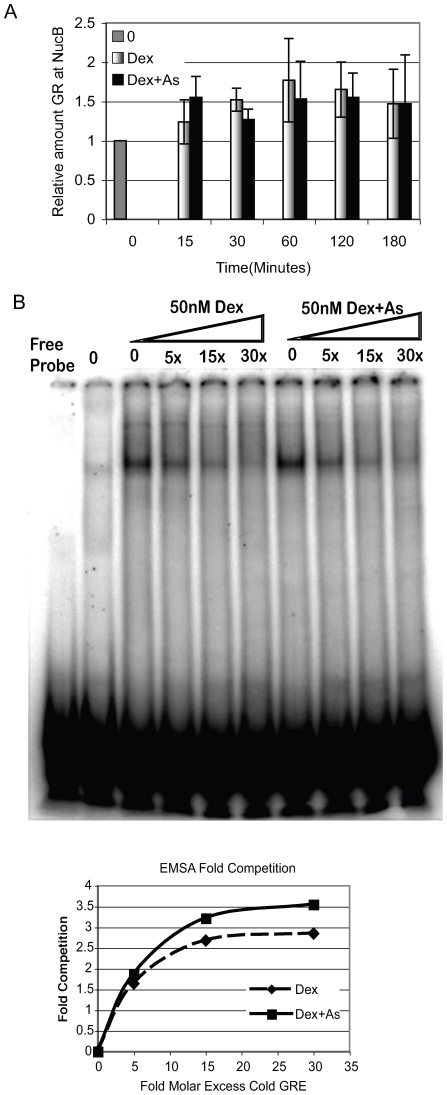
GR binds to the MMTV promoter equally in the presence of Dex±iAs. (A) ChIP analysis of GR interaction with NucB. Cells were treated with 5 nMDex±8 µM iAs and ChIP assays were done with antibody to GR. NucB bound by GR was quantified by qPCR or by conventional PCR. *n = 7–8* independent experiments. (B) EMSAs done with NEs from 1470.2 cells that were untreated (lane 2–0) or treated with 50 nM Dex±8 µM iAs for 30 minutes. [^32^P]-labeled consensus GRE±5, 15, and 30-fold molar excess unlabeled competitor GRE visualized as shifted band by PhosphorImager analysis. The Inset graph shows the competition quantified (shifted probe/(Free+shifted)) from each reaction shown.

GRs are predominantly cytoplasmic prior to ligand binding and translocate to the nucleus and to targeted GREs when ligand is bound to the receptor [24]. It was previously shown that low levels of iAs do not significantly alter GR translocation into the nucleus, but whether iAs affects GR binding to the GRE was not tested [7]. To determine if iAs affects GR/GRE binding, 1470.2 cells were treated with 5 nM Dex±8 µM iAs for 15, 30, 60, 120, or 180 min. Chromatin immunoprecipitation (ChIP) analysis was done to determine GR association with the MMTV promoter on nucleosome B (NucB) that has 4 GREs. GR was associated with NucB by 15–30 min of treatment with no detectable difference in cells treated with Dex±iAs (Fig. 2A). These data confirm that GR translocates to the nucleus, and binds to the MMTV GRE in the presence of iAs.

To determine whether iAs affects the DNA-binding kinetics of ligand-bound GR, electromobility shift assay (EMSA) competitions were done. Nuclear extracts (NEs) made from cells treated for 30 min with 50 nM Dex alone or 50 nM Dex plus 8 µM iAs were incubated with a radiolabeled consensus GRE [25] with 0, 5x, 15x, or 30x molar excess of unlabeled competitor GRE. 50 nM versus 5 nM Dex was used in this assay to allow detection of GR bound to the GRE. With 5 nM Dex fewer GRs enter the nucleus and bind to the GRE than with 50 nM resulting in a weak shifted signal. GR was competed from the radiolabeled GRE with as little as 5x unlabeled competitor GRE and almost fully with 15–30x (Fig. 2B). The slope, based on values from quantification of the shifted bands, was almost identical with either treatment (see Inset). These data indicate that treatment with Dex±iAs does not significantly change the DNA binding characteristics of GR under conditions in which transcriptional activation is inhibited by iAs, and therefore DNA binding is an unlikely explanation for iAs-associated transcriptional repression from the MMTV promoter.

### Effects of iAs on GR-mediated Histone Modification

Activation and repression of transcription is accompanied by changes in histone PTMs and such modifications occur at the MMTV promoter on both histones H3 and H4 [20], [26]–[28]. Differences in histone PTMs in cells treated with Dex±iAs could indicate an iAs effect on the histone itself or on a protein with histone modifying activity, such as a coactivator or corepressor. ChIP assays were done with antibodies to specific acetylated or methylated amino acid residues and changes in modification at NucB on the MMTV promoter were determined. Because histone PTMs occur temporally, as has been demonstrated on the GR and PR-regulated MMTV promoter [26], [28] and on the estrogen receptor-regulated pS2 promoter [29] all experiments were done in a time course as in Fig. 1E.

**Figure 3:**
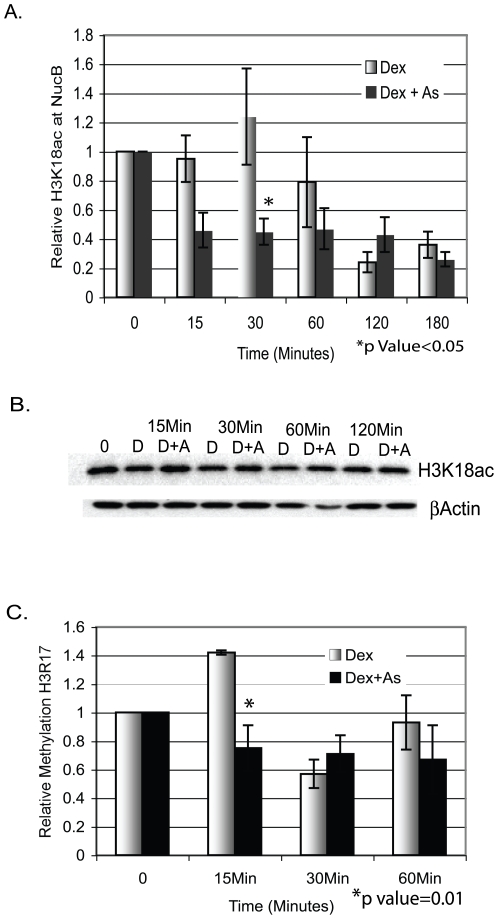
iAs inhibits histone H3K18 acetylation and H3R17 methylation. (A) 1470.2 cells were treated with 5nM Dex±8 µM iAs for the indicated times and ChIP assays were done with an antibody to H3K18ac. H3K18ac at NucB represents qPCR values from primers/probe to NucB corrected for Input DNA, expressed as a ratio of treated to untreated cells (0) arbitrarily set to 1. *n = 4–5* independent experiments. (B) Western blot analysis of NEs from cells treated as above with antibody to H3K18ac and b-actin. (C) 1470.2 cells treated with 5 nM Dex±8 µM iAs as above and ChIP assays were done with an antibody to H3R17me. qPCR was done with primers/probe to NucB and analyzed as above. *n = 3–5* independent experiments.

Following treatment with 5 nM Dex±8 µM iAs there were no significant differences between treatments in acetylation at histone H4, at amino acids H4K5, H4K8, or H4K12 or on histone H3 at H3K14 and H3K4 (data not shown). These amino acids were targeted because they have been shown by others to undergo PTM changes upon activation of transcription. Histone H3K18 acetylation (H3K18ac) occurs with H3R17 methylation (H3R17me) on the estrogen-regulated pS2 promoter [19] and H3R17 is methylated at the MMTV promoter in response to GR activation [20]. We found that H3K18ac was inhibited by treatment with Dex+ iAs by 15 to 30 min (Fig. 3A). The increase in H3K18ac in response to Dex alone was just slightly higher than basal levels. In contrast, cells treated with Dex + iAs showed no increase in acetylation at 15 or 30 min. but instead a significant decrease relative to basal levels and importantly, relative to levels seen with Dex alone. At the estrogen-responsive pS2 promoter, H3K18ac increases early in activation and decreases significantly with time and transcriptional repression [19] similar to what is shown here at the GR-responsive MMTV promoter. Thus, H3K18ac associated with steroid hormone-mediated transcription, is disrupted by iAs. Acetylation differences did not occur globally but were promoter-specific, an important distinction because iAs does not inhibit transcription from all promoters. Additionally, the decrease in H3K18ac was not due to histone H3 loss (Fig. 3B).

To determine whether H3R17me correlates with H3K18ac in response to activation by GR, cells were treated with 5 nM Dex±8 µM iAs. H3R17me increased by 15 min of treatment with Dex alone, but not in the presence of iAs (Fig. 3C). Together, the decrease in H3K18ac and H3R17me in cells treated with Dex + iAs versus Dex alone suggests that iAs-mediated inhibition of transcription may, at least in part, be due to changes in histone modification.

### CBP/p300 at the MMTV Promoter

**Figure 4:**
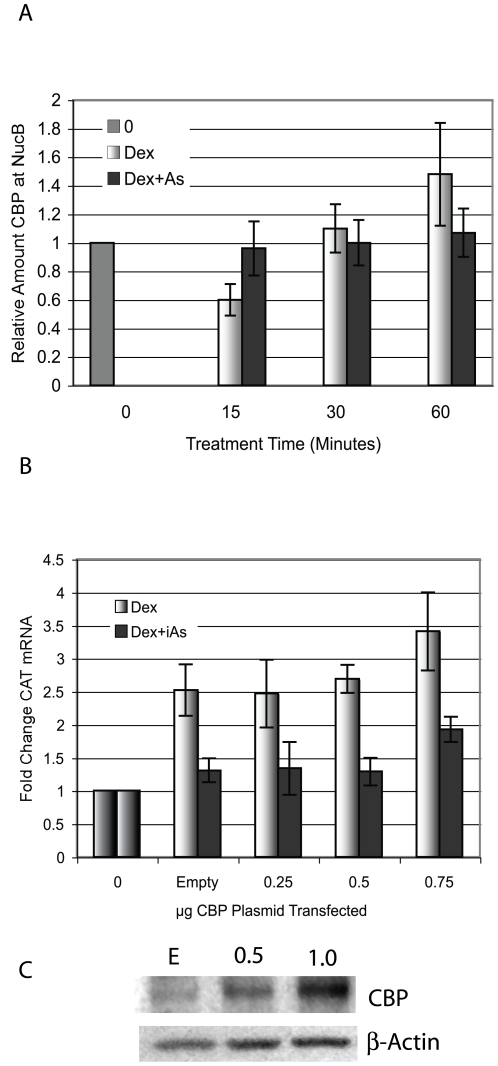
CBP binds to the MMTV promoter in the presence of iAs but does not restore iAs-repressed transcription. (A) ChIP assay in 1470.2 cells with an antibody to CBP. Treatment was with 5 nM Dex±8 µM iAs for the times indicated. qPCR was done with primers to NucB and values were corrected for Input DNA and expressed as a ratio of treated to untreated (0) arbitrarily set to 1. *n = 9* independent experiments. (B) Cells were transfected with pcDNA3-CBP-Flag or Empty vector at indicated concentrations and treated with 5 nM Dex±8 µM iAs for 24 h. Zero is untreated baseline activity. CAT mRNA was measured by RT-qPCR corrected for b-actin mRNA. The values indicated on the y-axis are fold changes in the RT-qPCR Units. *n = 4* independent experiments. (C) Empty vector (E) or pcDNA3-CBP-Flag was transfected at the concentrations indicated (µg) and western blot analysis was done to determine CBP protein over-expression.

Both CBP and p300 are protein acetyltransferases that interact with the MMTV promoter [28], [30] and can acetylate H3K18 in association with transcriptional activation at steroid hormone regulated promoters [19], [27]. Because H3K18 is less acetylated in the presence of iAs than in cells treated with Dex alone, these proteins became candidate iAs targets. Both proteins are post-translationally modified by cell signaling pathways and the PTMs can affect their enzymatic activity or their interaction with the the promoter via p160 coactivators, SRC1, GRIP1/SRC2, or AIB1/SRC3 [31], [32]. To determine whether iAs inhibits CBP interaction with the MMTV promoter at NucB, ChIP assays were done after cells were treated with 5nM Dex±8 µM iAs (Fig. 4A). No treatment-specific differences were found in promoter association by CBP. ChIP experiments were also done with antibody to p300 with a similar result (data not shown).

To determine whether over-expression of CBP could restore transcription in cells treated with Dex+iAs, cells were transfected with an expression plasmid for CBP and after recovery were treated for 24 hours with 5 nM Dex±8 µMiAs. Over-expressed CBP was unable to restore transcription in iAs-treated cells when compared to transcription seen with Dex alone (Fig. 4B). Thus, although H3K18 is not acetylated with iAs treatment there was no apparent difference in the presence of CBP at NucB and over-expression did not restore transcription. Over-expression of p300 was also unable to restore iAs-mediated transcriptional repression (data not shown). Together these data suggest that iAs may inactivate the enzymatic activity of either CBP, p300 or both proteins because although they are associated with the promoter, one of their targets, H3K18 is not acetylated when iAs is present.

### iAs Disrupts CARM1 but not GRIP1 Interaction with the MMTV promoter

**Figure 5:**
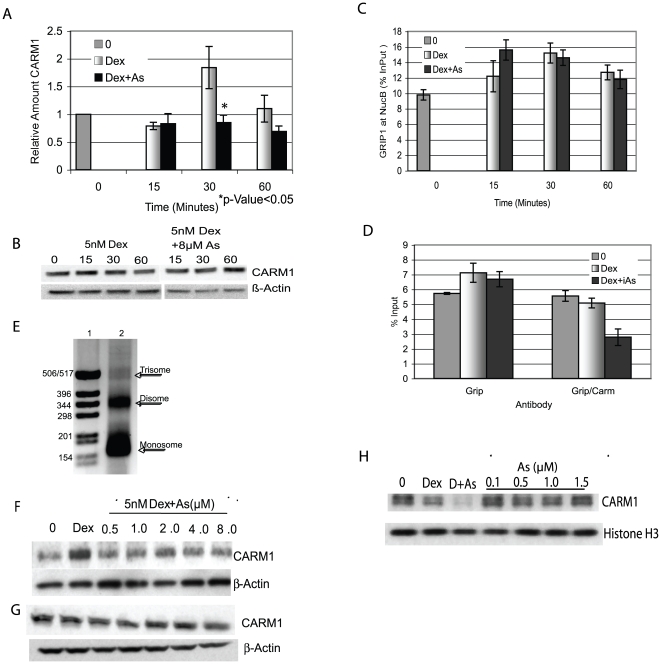
CARM1 but not GRIP1 interaction with the MMTV promoter is disrupted by iAs. (A) ChIP assay with an antibody to CARM1 of 1470.2 cells treated with 5 nM Dex±8 µM iAs. qPCR was done with primers to NucB and values were corrected for Input DNA and expressed as a ratio of treated to untreated (0) arbitrarily set to 1. *n = 4–6* independent experiments. (B) Western blot analysis of NEs treated as above incubated with antibodies to CARM1 or b-actin. (C) ChIP assays and analysis as in (A) done with an antibody to GRIP1. Experiment shown is representative of 3 independent experiments. *n* = 3 replicates. (D) Sequential ChIP analysis with antibodies to GRIP1 (first Ip) and CARM1 (second Ip). qPCR primers were to NucB and values expressed as NucB/InPuts (%InPut). Experiment shown is representative of 4 independent experiments. *n* = 3 replicates. (E) CARM1 bound to the MMTV promoter decreases in the presence of iAs in an *in vitro* pull-down assay. Micrococcal nuclease digestion of an *in vitro* assembled MMTV with Nucs A–C. DNA was visualized by agarose gel electrophoresis. Lane1 = Molecular weight markers and Lane 2 = MNase digested MMTV assembled template. (F) *In vitro* assembled MMTV promoter (Nucs A–C) incubated with 60 µg NE from cells treated for 30 minutes with 5 nM Dex±iAs from 0.5 to 8 µM. Proteins bound to the MMTV *in vitro* template were separated by SDS-PAGE followed by western blot analysis with antibodies to CARM1 or to b-actin. A representative experiment repeated twice is shown. (G) Western blot analysis of NEs treated as in (F) incubated with antibodies to CARM1 or b-actin. (H) NEs from cells treated with 5 nM Dex incubated with the *in vitro* template and exogenously added iAs from 0.1 to 1.5 µM. Western blot of MMTV-associated proteins with antibody to CARM1. Lane 0 = NE from untreated cells, Lane “Dex” = NE from 5 nM Dex treated cells and Lane “D+As” is NE from cells treated with 5 nM Dex+8 µM iAs. Below is the same blot incubated with antibodies to histone H3. Representative of an experiment repeated twice.

CARM1 is a protein methyltransferase (PRMT 4) that specifically targets H3R17 for methylation [17] and acts synergistically with p160 coactivators to enhance transcription from steroid-regulated promoters including ER and GR [20], [21]. Because H3R17 was less methylated in response to iAs, CARM1 could either be displaced from the MMTV promoter or be at the promoter but not be enzymatically active. To distinguish between these possibilities, cells were treated with Dex±iAs and ChIP analysis was done with an antibody to CARM1. By 30 min of treatment with Dex alone CARM1 was at the promoter but was not when iAs was present (Fig. 5A). Western blot analysis of NEs confirmed that CARM1 was available for binding and not degraded after iAs treatment (Fig. 5B). Thus, iAs inhibits CARM1 interaction at the GR-activated MMTV promoter which can account for the lack of H3R17me. ChIP assays also showed that CARM1 interaction with the SGK promoter was inhibited by iAs, similarly to the MMTV promoter (data not shown).

The p160 coactivator GRIP1 interacts with GR and CARM1 interacts with the promoter by binding to the C-terminal domain of GRIP1 [33]. To determine if iAs affects GRIP1 interaction with GR, cells were treated with Dex±iAs and ChIP assays were done with antibody to GRIP1. There was no difference in the amount of GRIP1 at NucB with iAS treatment (Fig. 5C). These data suggest that iAs affects the CARM1/GRIP1 interaction but not the GRIP1/GR interaction at the times tested. A sequential ChIP experiment was done to test whether the CARM1/GRIP1 interaction was disrupted by iAs. Cells were treated with 5 nM Dex±8 µM iAs for 30 min and ChIP assays were done with antibody to GRIP1 followed by antibody to CARM1. In the first step, GRIP1 was found at NucB with Dex±iAs (Fig. 5D) as seen in Fig. 5C. The GRIP1 immunoprecipitated material was then incubated with antibody to CARM1 to determine whether CARM1 was associated with GRIP1 at the promoter. The CARM1/GRIP1 interaction was intact in the presence of Dex alone but was not in the presence of Dex + iAs (Fig. 5D).

*In vitro* pull-down assays further tested CARM1 interaction with the MMTV promoter in response to Dex + iAs (Fig. 5E–H). The assay utilizes a short DNA fragment (Nucs A–C) of the MMTV promoter assembled into a regularly spaced nucleosomal array coupled to a magnetic bead (Fig. 5E). The *in vitro* MMTV template was incubated with NE isolated from cells treated with Dex±iAs at concentrations that inhibit transcription in these cells (Fig. 1B). CARM1 bound to the promoter with Dex alone, but even at the lowest concentration of iAs tested the amount of CARM1 bound did not exceed that in NEs from untreated cells (zero) (Fig. 5F). CARM1 seen in the untreated cells can be attributed to the low level of background activation by GR resulting from a small amount of GR in the nucleus before treatment (see EMSA-Fig. 2B-Lane 2). NEs used in the pull-down reactions show an approximately equal amount of CARM1 was present in all of the NEs (Fig. 5G). These *in vitro* data confirm results from the ChIP analysis (Fig. 5A) and support the conclusion that CARM1 interaction with the MMTV promoter is inhibited by iAs.

To determine whether the inhibition of CARM1 interaction with NucB was a direct or an indirect effect of iAs on CARM1, cells were treated with Dex alone and NE from the cells was incubated with the *in vitro* MMTV DNA. iAs was added directly to the *in vitro* reactions at concentrations equivalent to those found in the nucleus after treatment of cells with 5 nM Dex + 8 µM iAs for 15 to 30 minutes as previously determined by Inductively Coupled Plasma Mass Spectrometry (ICP-MS) analysis of NEs (data not shown). There was no decrease in CARM1 bound at any concentration of iAs added (Fig. 5H) as was seen when cells were treated with iAs (labeled “Dex” and “D+As”). These data suggest that iAs does not have a direct effect on CARM1 but is acting indirectly to disrupt the CARM1/GRIP1 interaction.

### Over-expression of CARM1 restores iAs-inhibited transcription

**Figure 6:**
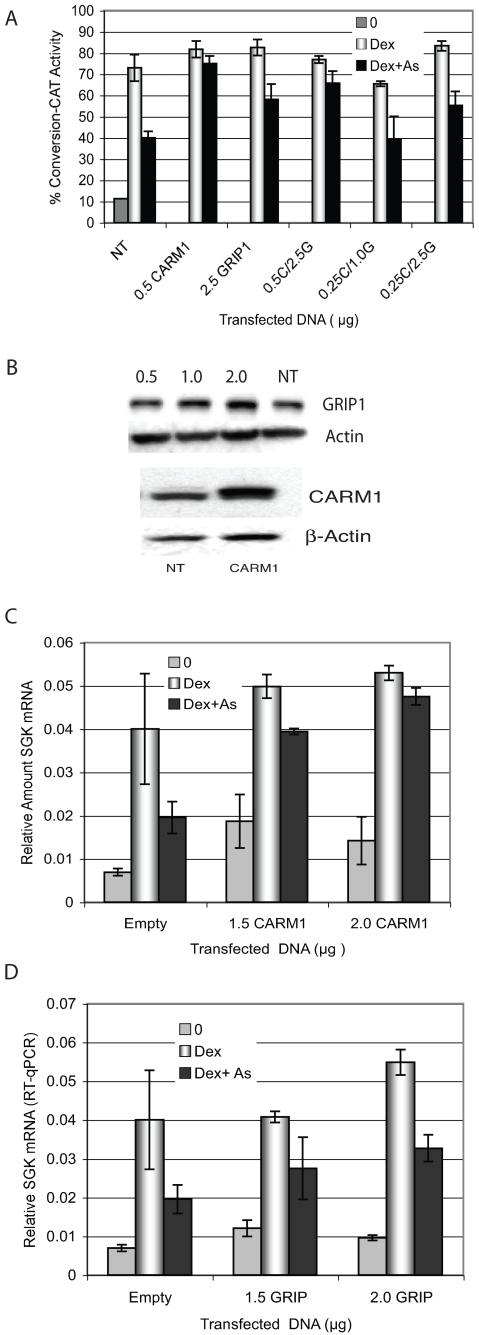
Over-expression of CARM1 restores iAs-mediated transcriptional repression. (A) Cells were transfected with pSG5-HA-CARM1, pSG5-GRIP1/FL or a combination of the two expression plasmids at indicated concentrations and treated with 5 nM Dex±8 µM iAs for 24 h. CAT activity was measured and expressed as percent conversion from unacetylated to acetylated chloramphenicol. Representative of an experiment repeated 3 times *n* = 3 replicate points. (B) Western blot analysis of proteins from cells transfected with 0.5 µg of pSG5-HA-CARM1 or pSG5-GRIP1/FL plasmid or non-transfected cells (NT), incubated with antibodies to CARM1, GRIP1 and b-actin. The HA-tagged CARM1 migrates slightly higher in the gel than endogenous CARM1. (C) Cells transfected with pSG5-HA-CARM1 at the indicated concentrations or pSG5-HA-EMPTY at 1.5 µg and treated for 2 hours with 5 nM Dex±8 µM iAs. SGK mRNA was measured by RT-qPCR corrected for actin mRNA. The values indicated on the y-axis are RT-qPCR Units. Experimental points are in triplicate and the experiment was repeated twice. (D) pSG5-GRIP1/FL or Empty vector was over-expressed in cells at the concentrations indicated and SGK mRNA was measured by RT-qPCR and analyzed as in (C).

If the decrease in CARM1 promoter interaction is functionally associated with the decrease in transcription due to iAs then over-expression could overcome repression and restore transcription. CARM1 was over-expressed and cells were treated with Dex±iAs. CAT activity from the stably integrated MMTV-CAT reporter was about 35% less in non-transfected (NT) cells treated with Dex+iAs compared to Dex alone (Fig. 6A). In cells transfected with 0.5 µg CARM1, activity was restored in the iAs-treated cells to the same levels as cells treated with 5 nM Dex alone. Because CARM1 interacts with the promoter via GRIP1, GRIP1 was also over-expressed to determine whether it could restore iAs-repressed transcription. If over-expressed GRIP1 was able to restore transcription it would raise the possibility that GRIP1 is also a target for iAs. CAT activity in iAs-treated cells was slightly higher than in similarly treated non-transfected (NT) cells, but was not fully restored as with CARM1 over-expression. If 0.5 µg CARM1 was over-expressed with GRIP1, CAT activity was restored in iAs-treated cells, but co-expression of 0.25 µg CARM1 (a concentration that does not restore CAT activity) with 0.1 or 2.5 µg GRIP1 did not restore CAT activity. Western blot analysis showed both CARM1 and GRIP1 were over-expressed in transfected cells (Fig. 6B).

Transcription from the SGK promoter was inhibited by iAs treatment similarly to that at the MMTV promoter (Fig. 1D & E). To determine if CARM1 or GRIP1 are functionally involved in iAs-mediated transcriptional repression from the endogenous SGK promoter, either CARM1 or GRIP1 were over-expressed and SGK mRNA was quantified by qRT-PCR. As with CAT activity, SGK mRNA expression was restored when CARM1 was over-expressed (Fig. 6C). A somewhat higher level of transcription was observed in the presence of iAs when GRIP1 was over-expressed but not to the extent seen with CARM1 (Fig. 6D). These data suggest that the decrease in transcription by iAs is functionally related to the absence of CARM1 from the promoter because over-expression restores GR-mediated activation. We cannot discount that GRIP1 has a role in iAs-mediated transcriptional repression from these data because there is less of an iAs effect when it is over-expressed that is consistently seen in the over-expression experiments.

## Discussion

### Inhibition of Transcription Initiation by iAs

Although we see little difference in initiated transcripts after 60 minutes of treatment with 5 nM Dex + 8 µM iAs compared to Dex alone (Fig. 1C) we see a significant difference by 2 hours. This suggests that iAs represses transcription through an effect on initiation. The data from the REAA assays (Fig. 1D) in which iAs inhibits GR-mediated chromatin remodeling lend more strength to this hypothesis. The decrease in chromatin accessibility in the presence of iAs suggests an effect on the chromatin remodeling machinery. ATP-dependent chromatin remodeling complexes found at steroid hormone receptor-regulated promoters include the SWI/SNF-related BRG1 and brahma (BRM) ATPases. Both are recruited to GR and other steroid receptor-activated promoters [34]–[37]. Interestingly, BRG1 and CARM1 have been co-purified in a complex, **N**ucleosomal **M**ethylation **A**ctivator **C**omplex (NUMAC) on an ER-target gene where they physically interact with each other [38]. Furthermore, binding of BRG1 to the late myogenic gene promoters is facilitated by CARM1. If CARM1 is not present, BRG1 is not found at these promoters [39]. Data presented here show that at the MMTV promoter there is decreased chromatin remodeling in the presence of iAs and CARM1 is not present at the promoter. These data suggest that BRG1 or another ATP-dependent chromatin remodeling factor may not be associated with the promoter. How iAs inhibits chromatin remodeling at the MMTV promoter is currently under investigation.

### Coactivator interactions

GRIP1 was found at NucB on the MMTV promoter following Dex + iAs treatment but CARM1 was not which suggested that iAs may inhibit transcription by disrupting the CARM1-GRIP1 interaction (Fig. 5C). Furthermore, in a sequential ChIP assay in which GRIP1 was first immunoprecipitated followed by CARM1, the GRIP1-CARM1 interaction was disrupted by iAs. Additionally, over-expression of CARM1 restored iAs-repressed transcription, Together these data strongly suggest that CARM1 is a likely iAs target in the cell. GRIP1 was at the promoter in cells treated with iAs, and over-expression was able to partially restore transcription. Thus we cannot eliminate the possibility that GRIP1 is a target for iAs. A problem we encountered in the GRIP1 over-expression experiments was that we could achieve only small increases in GRIP1 protein by over-expression despite seeing significant increases in GRIP1 mRNA (data not shown). All three of the p160 coactivator proteins are regulated by phosphorylation-dependent ubiquitin-proteasomal pathways [40]–[43] which is likely why there is a limited amount of GRIP1 protein over-expression relative to the increase in mRNA in these cells. Because GRIP1 is at the promoter in the presence of iAs and it interacts with the MMTV promoter via GR [44], the GR-GRIP1 interaction must be intact. An effect of GRIP1 over-expression on transcription would also depend on the stability of the GR-GRIP1 interaction. If the interaction is very stable and there is little turnover, then GRIP1 over-expression may not be effective in replacing the GRIP1 already there. This could account for the very small increase in transcription when GRIP1 is over-expressed and leaves open the possibility that GRIP1 is an iAs target.

The GRIP1-CARM1 interaction domain on either protein may be a target for iAs resulting in CARM1 dissociation from GRIP1, or alternatively, another protein that stabilizes the CARM1-GRIP1 interaction could be an iAs target. The first possibility would be consistent with iAs mediated changes in interaction domain PTMs that affect CARM1-GRIP1 protein-protein interaction. CARM1 associates with promoters by binding to the C-terminal AD2 domain of GRIP1/SRC2 via CARM1s central domain [45] and PTMs on the related p160 coactivator SRC3/AIB1/p/CIP, including phosphorylation and methylation mediated by cell signaling pathways, affect coactivator complex assembly and stability on ERs [46]–[48]. Interestingly, there are three predicted sites for p38MAPK-mediated phosphorylation in the AD2 domain of GRIP1 where CARM1 binds [49]. iAs has stimulatory and inhibitory affects on multiple signaling pathways including p38MAPK [50] as well as extracellular signal-regulated kinases (ERK1/2), c-Jun terminal and stress activated kinases JNK/SAPKs [51], and mitogen-activated protein kinases (MAPKs), (MSK1)[52]. These pathways have the potential to post-translationally modify either CARM1 or GRIP1 [53], [54]. If the interaction between the two proteins is destabilized rather than completely inhibited by iAs, an increase in the concentration of either protein could change the equilibrium characteristics in the nucleus and potentially drive enough of an interaction to restore transcription. The experiments in which transcription increased in parallel with CARM1 over-expression support destabilization of the CARM1-GRIP1 interaction by iAs (Fig. 6A & C). The data also support an indirect effect of iAs on CARM1 (Fig. 5H) which would be consistent with inappropriate PTMs on either CARM1 or GRIP1 mediated by the disruption of a cell signaling pathway. It is also possible that another protein that stabilizes the CARM1-GRIP1 interaction is an iAs target. For example TIF1a/*Trim24* forms a complex with CARM1 and GRIP1 and its role may be in stabilizing the CARM1-GRIP1 interaction [55] making TIF1a a candidate iAs target in these cells.

The stability of CARM1 at promoters may also be affected by acetylation of H3K18 and H3K23 [19]. The significant decrease in H3K18ac in response to iAs exposure by 15–30 minutes (Fig. 3A) is likely due to the inactivation of a promoter-bound acetyltransferase such as CBP or p300, both of which target H3K18 for acetylation [19], [27]. CBP associates with the MMTV promoter equally well in cells treated with Dex±iAs (Fig. 4A) as does p300 (data not shown) which makes inactivation of histone acetyltransferase (HAT) activity a likely possibility. Thus, if the stability of CARM1 at the promoter is dependent on H3K18ac, the inactivation of CBP HAT activity may be a critical event in iAs-mediated inactivation of transcription and would almost certainly affect other aspects of the activation process [56]. Ongoing studies will determine whether iAs inhibits HAT activity of CBP and post-translational modifications on CBP or p300 that can influence HAT activity to begin to determine their role in transcriptional repression by iAs.

### iAs-mediated Inhibition of Histone Modification

The apparent level of H3K18ac and H3R17me in response to iAs in these experiments was below basal levels (Fig. 3). It is possible that a demethylase (H3R17) or a histone deacetylase (H3K18) is inappropriately associated with the promoter. Notably, nickel and chromium, two genotoxic metals, repress transcription rapidly and do so partially through mechanisms that affect histone modification [57]–[59]. However, there are multiple copies of MMTV-CAT stably integrated in the cell line used here and there is basal transcription from the MMTV promoter before the cells are treated. This would be associated with some H3K18 acetylation and H3R17 methylation. The small increase in H3K18ac and H3R17me compared to basal levels following treatment with Dex (Fig. 3) is likely because basal levels of modification are high which minimizes the amount of detectable increase with Dex treatment. Likewise, when iAs is present and histone PTMs are inhibited, basal transcription would also be inhibited. This is apparent in the REAA assay shown in Fig. 1D where chromatin remodeling is inhibited to below basal levels by iAs. Furthermore, it has been demonstrated that in a related cell line there is a population of open (active) chromatin templates in the basal state and some inactive or closed chromatin templates in the activated state [60]. Thus, we think it less likely that a deacetylase is responsible for decreased H3K18ac due to iAs than to inhibition of an acetylase activity, for example by CBP or p300.

### A mechanism common to iAs-mediated transcriptional inhibition at other promoters?

To date the mechanisms that underlie iAs-mediated disruption of transcription by steroid hormone receptors have not been characterized, although it is well documented that transcription by these highly related receptors is disrupted by exposure to iAs in the low micromolar range [7]–[10]. Because iAs can bind to vicinal thiols which are found in the steroid binding domain and the DNA binding domain of steroid hormone receptors [61], two possible hypotheses are that iAs might inhibit transcription by interfering with either steroid binding to receptor or receptor binding to DNA. In fact Simons et al [62] showed that in an *in vitro* system 7 µM iAs was effective in inhibiting Dex binding to GR. However, this concentration of iAs is not an achievable intracellular concentration because it would be toxic and result in cell death. In our analyses by ICP-MS of intracellular As^+3^ we found that treatment of cells with 8 µM iAs for 15 minutes to 1 hour, resulted in only 0.1 to 1.5 µM intranuclear As^+3^ with a correlation between the length of time of treatment and the amount of As^+3^ detected (data not shown). Note that these are the concentrations used in the *in vitro* system to determine whether iAs is acting directly or indirectly on proteins at the promoter (Fig. 5H). Treatment of 1470.2 cells with higher iAs concentrations than 8 µM induces a stress response and results in cell death (data not shown). Others have shown that GR does in fact translocate from the cytoplasm to the nucleus in the presence of iAs at similar concentrations to those used in this study [7]. This transition is ligand dependent and it is shown here that GR binds to a GRE equally well in cells that have been treated with iAs at levels that inhibit transcription (Fig. 2). Thus it appears that netiher iAs-mediated inhibition of steroid hormone binding to GR or GR binding to the promoter are likely mechanisms underlying inhibition of transcription by iAs.

Because both CARM1 and one of the p160 coactivators (SRC1, GRIP1/SRC2/TIF2, or SRC3/AIB1/p/CIP/TRAM) are essential to transcriptional regulation at all steroid hormone receptor-regulated promoters, identification of CARM1 and GRIP1 as players in iAs-mediated transcriptional inhibition raises the possibility that iAs may repress transcription by other steroid hormone receptors similarly. In support of this, we have found that iAs-mediated inhibition of an estrogen-responsive promoter in MCF7 breast cancer cells is functionally related to iAs effects on CARM1 and on SRC3/AIB1 a GRIP1 homologue that interacts with ERs (manuscript in preparation). While this manuscript was in preparation it was reported that TIF2, the human homologue of GRIP1/SRC2, does not bind to an AR-regulated promoter in LNCaP cells 24 hours after treatment with arsenic trioxide (As_2_O_3_/ATO) and an androgen [63]. It was also shown that AR was no longer at the promoter 24 hours after ATO treatment however, and p160 coactivators interact with hormone-responsive promoters via interaction with steroid receptors so TIF2 would not be expected to be there. No direct evidence to support iAs-mediated disruption of the AR/TIF2 interaction is shown. Our data shows that iAs inhibits CARM1 but not GRIP1/SRC2 interaction with the GR-activated MMTV promoter as early as 30 minutes following treatment with Dex and iAs in the form of NaAsO_2_. It is possible that ATO has a different affect on coactivator interaction than NaAsO_2_ but this is unlikely since both forms are sources of trivalent inorganic arsenic (As^3+^). It is also certain that after 24 hours of treatment the promoter-associated proteins would be very different than at 30 min after treatment. Because of the significant differences in the experimental approaches used it is difficult to determine from these data whether the dynamics of GRIP1/TIF2 interaction are the same or different in response to iAs and steroid hormone at GR and AR-regulated promoters.

CARM1 and p160 coactivator family members are also part of the transcription complex with some non-steroid-regulated transcription factors including p53 [64]. Thus the disruption of CARM1 promoter interaction associated with exposure to low levels of iAs could potentially extend beyond steroid hormone-regulated gene expression. This would increase the potential for deleterious physiological effects on virtually every metabolic system by iAs and would further explain how iAs exposure can be associated with the multiplicity of diseases that it is.

Long term exposure to iAs is associated with many different diseases but is also used as a cancer therapeutic, primarily in the form of ATO. Interestingly, ATO inhibits the interaction of the corepressor SMRT with the fusion protein promyelocytic leukemia-retinoic acid receptor-a (PML-RARa) contributing to possible mechanisms in iAs-mediated remission in acute promyelocytic leukemia (APL) [65], [66]. AIB1/SRC3, is an oncogene, amplified in breast, prostate, pancreatic, and other cancers [67] and CARM1 is over-expressed in grade-3 breast cancer tumors [68]. CARM1 and p160 family members have been suggested as potential targets in cancer therapy. We suggest that some of the known therapeutic effects of iAs may be related to an effect on the CARM1-GRIP1 interaction demonstrated here. iAs inhibition of the GRIP1-CARM1 interaction could be beneficial if these proteins are inappropriately over-expressed as in some cancers, but iAs could also lead to disease if it disrupts the normal function of GRIP1-CARM1 interaction. Data presented here provide strong evidence that iAs disruption of transcriptional coactivator function is a key piece in iAs-mediated repression of steroid hormone-regulated gene transcription. It will be important to further characterize the mechanism of iAs-mediated disruption of the CARM1-GRIP1 interaction and to identify how other proteins such as CBP/p300 are involved in iAs-mediated transcriptional repression to more fully understand how iAs can be both detrimental to health and also be an effective therapeutic.

## Materials and Methods

### Cell Culture

The 1470.2 cell line derived from the mouse adenocarcinoma parent line, C127i, constitutively expresses GR and has multiple copies of stably integrated MMTV-chloramphenicol acetyl transferase (MMTV-CAT) [69]. Cells were grown in DMEM (Gibco) with either 10% calf serum (CS), or charcoal stripped 1% CS for 16–24 h prior to treatment with Dex (Steraloids, Newport, RI)±sodium arsenite (NaAsO_2_) (ScienceLab.com).

### Chromatin Immunoprecipitation (ChIP) Assays

Cells were cross-linked with 1.5 mM Ethylene Glycol-bis (Succinimidylsuccinate) (EGS) (Pierce, Rockford, IL) at 25°C for 25 min followed by 1.0% formaldehyde at 25°C for 10 min (GR, CARM1, GRIP1) or with formaldehyde only (Histone PTMs). Nuclei were isolated, and DNA digested to predominantly monosomes with micrococcal nuclease (0.0375 U/µg nucleic acid) (Roche Applied Science, Indianapolis, IN) 37°C for 6 min. ChIP was as described by the Upstate Biotechnology ChIP protocol (Upstate Millipore.com). Incubation with specific antibody or non-immune IgG was overnight at 4°C, protein A sepharose or magnetically coupled Protein G beads (Dynal/InVitrogen) were used to isolate immune complexes. In Sequential ChIPs antibody-antigen complexes from the first Ip were eluted by incubation in 10 mM DTT for 30 min at 37°C. Samples were diluted in 20 volumes 1% triton X-100, 2 mMEDTA, 150 mM NaCl, 20 mM Tris 8.0 and antibody added. Wash buffers were High salt (500 mM NaCl, 50 mM Tris 8.0, 1%NP-40, 0.1% SDS, 1 mM EDTA), Low Salt (150 mM NaCl, and as above with the addition of 0.5% Deoxycholate), LiCl wash (250 mM LiCl, and as above). Elution from beads was in 50 mM NaCl, 10 mM Tris 7.5, 5 mMEDTA with 0.5% or 1.2% SDS for 15minutes at room temp. DNA was purified with MinElute columns (Qiagen) and quantified by fluorimetry with PicoGreen (Molecular Probes, Eugene, OR) on a Synergy HT plate reader (Biotek, Winooski,VT) or by NanoDrop spectrophotometer (ThermoScientific, Fisher). Non-immune rabbit IgG was used to determine non-specific antibody interactions and was subtracted from specific interactions. Primers to an unrelated genomic region, ribosomal protein L30 (RPL30) (Cell Signaling Technology, Danvers,MA) or to 5S ribosomal DNA were used to determine that the changes seen were specific to the promoter.

### PCR Protocols

ChIP DNA was analyzed by quantitative real-time (qPCR) with a TaqMan MGB Probe with a 6-FAM reporter and primers to MMTV Nuc B (Table S1) (Applied Biosystems, Foster City, CA) on a MJ Research Chromo4 RealTime PCR Detector DNA Engine (Bio-Rad, Hercules, CA). qPCR values were quantified by Absolute Standard Curve Method using plasmid pM25 (MMTV promoter) [69], corrected for Input DNA (NucB/Corresponding Input Value) and expressed as a ratio relative to untreated cells (arbitrarily set to 1). Other mRNAs were quantified using SyberGreen (iQ SyberGreen SuperMix, BioRad) and normalized to b-Actin mRNA by the Comparative C(t) method [70] after cDNA was made, referred to as reverse transcibed quantitative PCR (qRT-PCR). cDNA for PCR was synthesized with MuLV reverse transcriptase (NEB, Ipswich,MA), and Random hexamers (Roche, Nutley,NJ). See Table S1 for Primers and probes used in PCR.

### Antibodies

See Table S2-Antibodies

### Nuclear Extracts (NEs)

Mini-Dignam NEs were made [71]. Cells were lysed in Buffer A (10 mM HEPES, pH 7.9, 1.5 mM MgCl_2_, 10 mM KCl, 0.5 mM DTT), nuclei were isolated/extracted in Buffer C (20 mM HEPES, pH 7.9, 25% glycerol, 0.42 M NaCl, 1.5 mM MgCl_2_, 0.2 mM EDTA, 0.5 mM PMSF, 0.5 mM DTT) and dialysis was for 2 hr against Buffer D (20 mM HEPES, 7.9, 20% glycerol, 0.1 M KCl, 0.5 mM PMSF, 0.5 mM DTT, 5 mM MgCl_2_). Protein was measured by Bradford assay (BioRad, Hercules,CA).

### Electromobility shift assays (EMSA)

EMSAs included 20 µg of NE in Buffer D with 30–60 fmol of [^32^P]-end-labeled wtGRE, 5′-GATCCGGTacaATCtgtTCTA-3′ [25], and 0.2 µg poly(dI-dC).poly(dI-dC) (Roche, Nutley,NJ) in 20 µl reactions separated on 5% acrylamide/0.5X Tris/Borate/EDTA gels at 4°C. Imaging was by PhosphorImager analysis (Molecular Dynamics) and quantification by ImageQuant software.

### Gel Electrophoresis and Western Blot Analysis

Proteins were separated on 10% polyacrylamide gels Pierce (Rockford, IL) or NuPAGE Novex BisTris 4–20% gradient gels (Invitrogen) in recommended buffers, transferred to Immobilon-P PVDF membrane (Millipore) and visualized by HRP-based chemiluminescence imaged on film or on an Alpha Innotek FluoroChem 8900 (San Leandro, CA).

### Nuclear Run-ons

Isolated nuclei (0.5 mg) were resuspended in 100 µl (10 mM Tris pH 8.0, 5 mM MgCl_2_, 40% glycerol, 2.5 mM DTT). One hundred microliters of 2x reaction mix (10 mM Tris 8.0, 5 mM MgCl_2_, 200 mM KCl, 200 U/ml RNasin, 1 mMCTP,1 mM GTP, 2 mM ATP, 2 µM UTP, 5 mM DTT) was added with 120 µCi/200 µl reaction [^32^P]-UTP (3000 Ci/mmol) (ICN Pharmaceuticals, Costa Mesa, CA). and incubated for 45 min 30°C. RNA was extracted with TRI Reagent LS (Molecular Research Center, Cincinnati, OH) and equal counts were added to nitrocellulose filters (Schleicher and Schuell, Keene, NH) previously blotted with PCR-amplified CAT reporter DNA (1 µg of 1 kB coding region), an equal amount of pUC18 DNA for background subtraction, and 5 s DNA for normalization. Hybridization was at 42°C in 50% formamide, 5x SSPE, 3x Denhardt's, 100 µg/ml yeast tRNA and 0.5% SDS for 12–16 h. Visualization/Quantification was on a Storm Typhoon PhosphorImager and ImageQuant analysis (Molecular Dynamics, Sunnyvale, CA).

### Restriction Enzyme Accessibility Assay (REAA)

Nuclei were harvested from treated cells and a portion quantified after proteinase K digestion (InVitrogen). 50 µg nuclei were digested with 10 U/µg SacI, New England Biolabs, for 15 minutes at 37°C in 50 µl of 50 mM NaCl, 50 mM Tris pH 8.0, 1 mM MgCl_2_, 1 mM β-mercaptoethanol, 2.5% glycerol. The reaction was terminated with 5 vol. 10 mM Tris (pH 7.5), 10 mM EDTA, 0.5% SDS, 100 µg/ml proteinase K, and digested overnight at 37°C to extract genomic DNA. DNA was prepared for qPCR by Min-Elute columns (Qiagen) and quantified by NanoDrop spectrophotometer (ThermoScientific, Fisher). qPCR was with 15–30 ng total DNA and primers that flank the SacI site in NucB (see Table SI). A PCR product indicated chromatin inaccessibility because the SacI site was not accessible. Quantification of PCR product was by the Comparative C(t) method with normalization to β-Actin. Percent Accessibility = (1− (Treated/Untreated)) x100.

### Magnetic Bead DNA

A 1.8 kb Sph1/Nco1 fragment of the MMTV LTR from the pGEM3ZFM-LTRCAT plasmid that includes Nucs A-F of the MMTV promoter, was biotinylated (20 ug DNA fragment, 1x Klenow Buffer, 1 mM MgCl_2_, 50 µM each dTTP aS, dGTP aS, dCTP aS (Axxora, San Diego, CA), 18 µM Biotin-14-dATP (Invitrogen Carlsbad, California), 10 U Klenow (Invitrogen, Carlsbad, California) at 25°C for 15 min and attached to streptavidin-coated magnetic beads using the Dynabead Kilobase Binder Kit (Dynal Biotech, Lake Success, NY) in 200 µl Kit binding buffer as described by Fletcher et al [72]. Digestion with Ple1 left Nucs A–C attached to the bead.

### Chromatin Assembly

Bead DNA (Nucs A–C) was assembled with HeLa core histones using Active Motif's (Carlsbad CA) Chromatin ATP-dependent Assembly Kit that utilizes purified recombinant human chromatin assembly complex ACF, and histone chaperone NAP-1 with modifications. Post assembly, 3×200 µl washes were done in Wash 1: Storage Buffer (SB)(10 mM HEPES (pH 7.8), 10 mMKCl, 1.5 mMMgCl_2_, 0.5 mM EDTA, 10% glycerol 10 mM b-glycerophosphate, 0.1% NP-40, 1 mg/ml acetylated BSA (Sigma), 0.5% Sarkosyl). Wash 2: SB with 200 mM KCl. Wash 3: SB with 2 mg/ml BSA. Washes 1 and 3 were at 25°C and wash 2 at 4°C. Assembled DNA was stored in SB with protease inhibitor cocktail (Calbiochem, San Diego, CA) no more than 2 days at 4°C before use.

### Pull-Down Assay

Chromatin assembled bead DNA was washed twice in Pull-Down buffer (20 mM Hepes [pH 7.3], 50 mM NaCl, 10% Glycerol, 0.05 mM EDTA, 5 mM MgCl_2_, 0.1% NP-40, 1 mM DTT, 0.5 mM PMSF, 1∶100 protease inhibitor cocktail (Calbiochem, San Diego, CA) and resuspended at 0.01 ug/ul. Reaction components were 31 ul Pull-down buffer, 2 ul 50 mg/ml acetylated BSA (Sigma-Aldrich, St. Louis, MO), 0.5 ul poly (dI:dC). poly (dI:dC) (Pharmacia) 1.0 ug/ul), 60 ug Nuclear Extract, 0.05 ug bead DNA, Buffer D to 50 ul on ice in a 96 well round-bottom polypropylene plate (Costar). After incubation at 30°C for 15 min immobilized bead DNA was magnetically immobilized (Dynal Inc., Lake Success, NY) to remove reaction mix and 2X 50 ul washes in cold Pull-down buffer. Bead DNA-bound proteins were separated on denaturing 4–20% Tris-Glycine gels (Invitrogen Carlsbad, California), followed by Western Blot analysis.

### Plasmid Transfections

Over-expression plasmids were pSG5-HA-CARM1 and pSG5-GRIP1/FL [33], [44] or pcDNA3-CBP-Flag [73], [74]. Lipofectamine Plus (Invitrogen, Carlsbad, CA) was used as directed in 12-well or 6-well cluster plates. Cells recovered for 24 h and medium was replaced with 1% stripped serum (HyClone) in DMEM. 24–48 h post-transfection cells were treated with Dex±iAs for indicated times, were harvested and resuspended in 0.25 M Tris (pH 7.5) for CAT and Bradford protein assays (Bio-Rad, Hercules, CA) or lysed in Cell Lysis Buffer (20 mM Tris 7.5, 100 mM NaCl, 0.5% NP-40, 0.5 mM EDTA, Protease inhibitors (Roche, Nutley, NJ)) for western blot analysis. Alternatively, RNA was isolated with RNeasy columns (Qiagen) and cDNA prepared for qRT-PCR.

### Chloramphenicol Acetyltransferase (CAT) Assays

Five micrograms protein was incubated for 30 min at 37°C with [^14^C]-chloramphenicol (ICN, Costa Mesa, CA) and 4 mM acetyl CoA (Sigma-Aldrich, Nutley, NJ), extracted with ethyl acetate and CAT activity was measured by thin layer chromatography. Acetylated products were visualized on a Molecular Dynamics PhosphorImager and analyzed with ImageQuant (GE Healthcare Life Sciences, Piscataway, NJ). CAT activity was expressed as percent conversion (acetylated chloramphenicol/total chloramphenicol).

### Statistical Analyses

Data are expressed as means and *SEM*. Statistical significance was determined by two-tailed t-test assuming equal variances.

## Supporting Information

Table S1 PCR Primer sequences
(0.04 MB DOC)

Table S2 Antibodies
(0.00 MB DOC)
