# Supplementary material for: Disruption of Histone Modification and CARM1 Recruitment by Arsenic Represses Transcription at Glucocorticoid Receptor-Regulated Promoters
Source: PLoS One. 2009 Aug 26;4(8):e6766. doi: 10.1371/journal.pone.0006766 (PMC2727952; doi:10.1371/journal.pone.0006766)
Supplement: Table S1 — PCR Primer sequences (0.04 MB DOC) [file pone.0006766.s001.doc]

**Table S1 PCR Primer Sequences**

| **Gene/Site** | **PCR Primer Sequences** |
| --- | --- |
| **NucB**  Forward  Reverse  **NucB**  **Probe**  **Actin coding**  Forward  Reverse  **CATcoding**  Forward  Reverse  **SGKcoding**  Forward  Reverse  **CARM1**  Forward  Reverse  **GRIP1**  Forward  Reverse REAA Assay Forward  Reverse | 5’- GGTTACAAACTGTTCTTAAAACGAGGAT 3’  5’-CAGAGCTCAGATCAGAACCTTTGA-3’  5’FAM-CCAAACCAAGTCAGGAAACCACTTGTCTCA-MGB 3’  5’-GACGGCCAGGTCATCACTATT-3’  5’-AGTTTCATGGATGCCACAGG-3’  5’-TTATACGCAAGGCGACAAGGT-3’  5’-CCATCACAGACGGCATGATG-3’  5’-GAGGGAGCGCTGCTTCCT-3’  5’-CCCAAGGCACTGGCTATTTC-3’  5’-ATGCAGAGGTCCTGAAGA-3’  5’-TGACCACGATGCGGTCTGT-3’  5’-CCAGAAAACGCAAGGAATGTC-3’  5’-CCGTTTCTCAGTGCTCCTTTTG-3’  5’- GGTTACAAACTGTTCTTAAAACGAGGAT 3’  5’-CCTCTGGAAAGTGAAGGATAAGTGA-3’ |
