# Supplementary material for: Disruption of Histone Modification and CARM1 Recruitment by Arsenic Represses Transcription at Glucocorticoid Receptor-Regulated Promoters
Source: PLoS One. 2009 Aug 26;4(8):e6766. doi: 10.1371/journal.pone.0006766 (PMC2727952; doi:10.1371/journal.pone.0006766)
Supplement: Table S2 — Antibodies (0.00 MB DOC) [file pone.0006766.s002.html]

404 Not Found

# Not Found

The requested URL /pone\_files/2009/07/17/00086128/01/86128\_1\_supp\_1\_k404tw.doc was not found on this server.

---

Apache Server at one.plosjms.org Port 80
